# Trans-Scale Progressive Failure Analysis Methodology for Composite Materials Incorporating Interfacial Phase Effect

**DOI:** 10.3390/ma18153667

**Published:** 2025-08-04

**Authors:** Zhijie Li, Fei Peng, Jian Zhao, Sujuan Guo, Lefei Hu, Yu Gong

**Affiliations:** 1 Key Laboratory of AI-Aided Airworthiness of Civil Aircraft Structures, School of Aerospace Engineering and Applied Mechanics, Tongji University, Shanghai 200092, China; lizhijie@tongji.edu.cn (Z.L.);; 2 Beijing Institute of Astronautical Systems Engineering, Beijing 100076, China; 3 National Key Laboratory of Strength and Structural Integrity, Xi’an 710065, China; 4 Shanghai Institute of Aircraft Mechanics and Control, Shanghai 200092, China; sujuanguo@ecust.edu.cn; 5 Key Laboratory of Pressure Systems and Safety, Ministry of Education, East China University of Science and Technology, Shanghai 200237, China; 6 Chongqing Key Laboratory of Heterogeneous Material Mechanics, College of Aerospace Engineering, Chongqing University, Chongqing 400044, China; gongyu@cqu.edu.cn

**Keywords:** composite, trans-scale, interface equivalence, progressive failure

## Abstract

Fiber-reinforced resin matrix composites are generally composed of fibers and matrix with significantly different properties, which are non-uniform and anisotropic in nature. Macro-failure criteria generally view composite plies as a uniform whole and do not accurately reflect fiber- and matrix-scale failures. In this study, the interface phase effect between fiber and matrix has been introduced into the frame of trans-scale analysis to better model the failure process, and the equivalent mechanical property characterization model of the interface phase has also been established. Combined with the macro–micro-strain transfer method, the trans-scale correlation of the mechanical response of the composite laminates between the macro scale and the fiber, matrix and interface micro scale has been achieved. Based on the micro-scale failure criterion and the stiffness reduction strategy, the trans-scale failure analysis method of composite materials incorporating the interface phase effect has been developed, which can simultaneously predict the failure modes of the matrix, fiber and interface phase. A numerical implementation of the developed trans-scale failure analysis method considering interface phase was carried out using the Python and Abaqus 2020 joint simulation technique. Case studies were carried out for three material systems, and the prediction data of the developed trans-scale failure analysis methodology incorporating interface phase effects for composite materials, the prediction data of the Linde failure criterion and the experimental data were compared. The comparison with experimental data confirms that this method has good prediction accuracy, and compared with the Linde and Hashin failure methods, only it can predict the failure mode of the fiber–matrix interface. The case analysis shows that its prediction accuracy has been improved by about 2–3%.

## 1. Introduction

Fiber-reinforced resin matrix composites have been widely used in aerospace due to their high specific strength, high specific modulus, fatigue resistance and good designability. Failure analyses of composite materials and their structures is an important part of ensuring their safe service. Traditional composite material failure criteria, such as the Hill–Tsai failure criterion [1], Hashin failure criterion [2] and Tsai–Wu failure criterion [3], are macro-scale failure criteria that can effectively predict the overall response of composite materials at the macro scale. Many studies [4,5,6,7,8] have used macro-scale failure criteria to predict the damage failure behavior of composite materials and have achieved good accuracy. However, macro-scale criteria are difficult to employ to capture the microscale failure mechanisms of fibers, matrices and interface phases. Fiber-reinforced composite materials are usually composed of fibers and matrix, and the mechanical properties of fiber and matrix vary significantly, as well as the stress and strain levels of fibers and matrix on the micro scale when subjected to external loads. Consequently, the failure process of fiber and matrix phases cannot be accurately reflected by failure analyses based on the stress and strain data obtained by unifying the fiber and matrix. Further development of failure analysis techniques that reflect the relationship between the micro scale of fibers and matrix and the macro scale of layup is needed to better detect damage modes and capture the process of damage propagation in composite materials.

Trans-scale failure analyses of composite materials can be realized by correlating the macro and micro mechanical responses through the representative volume element (RVE). Gosse et al. [9] developed a method for obtaining strain magnification factors using representative volume elements of micro mechanics. Yudhanto [10] calculated the strain amplification coefficient using the three-dimensional finite element method. Tay et al. [11] used the strain-invariable failure criterion for micro mechanics analyses and discussed the effects of material properties and volume fraction on the magnification factor. Subsequently, Jin et al. [12] introduced the concept of a stress amplification factor, explicitly expressing the correlation between macroscopic and microscopic stresses in the mathematical equations based on the linear stress–strain relationship. Ha et al. [13] developed the Micro Mechanics of Failure (MMF) method, based on stress amplification factors, to predict the failure of continuous fiber-reinforced composites, where the micro-damage onset conditions of the fiber and matrix are independent. The macro stress can be transformed into the micro stress of the fiber and matrix by stress amplification factors, and the failure of laminate can be predicted using the MMF prediction criterion. Subsequently, Ha et al. [14] proposed a progressive damage model based on micro mechanics to predict the macroscopic failure behavior of composite laminates under multiaxial mechanical loads and thermal effects. Buchanan et al. [15] proposed a method for determining the micro-strain field, taking into account the effects of thermomechanical deformation. Sun et al. [16] developed a non-iterative element failure method and combined it with the MMF failure criterion, further studying and modifying the MMF criterion. Huang et al. [17,18] proposed a micro mechanics-based progressive failure analysis strategy for fiber-reinforced composites. Li et al. [19,20], based on the MMF theory, studied the initial failure, subsequent propagation, final failure and strength of open-hole compression structures of carbon fiber-reinforced polymer composites. Moreover, Li et al. [21] also used the stress amplification factors to calculate the stresses at key-points of the micro-scale model, the maximum stress failure criterion for fiber and the von Mises criterion for matrix to judge the failure, and studied the failure of composite laminate in open-hole tension. Wang et al. [22,23] developed a progressive failure method based on the MMF criterion and material performance degradation principle, and predicted the ultimate strength and complex failure behavior of composite containers under high pressure and thermal loads. Liao et al. [24] introduced the MMF criterion to establish the relationship between macroscopic and microscopic stresses through stress amplification factors, but did not consider the influence of temperature and conducted impact simulation experiments. Liu et al. [25] proposed a multi-scale analysis strategy to analyze the failure behavior of open-hole compression laminates, using MMF to construct macro–micro relationships, and studied the accuracy of the multiscale model in predicting strength and failure modes at different temperatures. Lou et al. [26,27] used stress amplification factors to calculate the microscopic stresses of fiber and matrix to study the compression behavior of impacted composite laminate, further considering interface failure, the multi-scale progressive damage model was validated using open-hole compression tests. Sun et al. [28] proposed a new multi-scale progressive damage model based on microscopic RVE, introducing a SAF (stress amplification factor) database to characterize the microscopic stress states of fiber and matrix. Lee et al. [29] predicted progressive failure behavior of woven composite structures under quasi-static mechanical loads via multi-scale (macro–meso–micro) simulations using the stress amplification factor. Zheng et al. [30,31,32,33] proposed a multi-scale damage model for MMF, introducing the SSAF (strain and stress amplification factor), establishing the transfer relationship between meso and micro stresses through the stress amplification factor, and using the SAF to convert meso stresses into stresses in the micro fiber and matrix for studying the complex failure behavior of three-dimensional woven composites.

Several researchers have also studied the interface between fiber and matrix [34,35,36,37]. They established representative volume elements comprising the fiber, matrix and interface, considering the random distribution of fiber. Interface damage is simulated using a cohesive model, and a bilinear traction–separation law is adopted. These studies establish RVEs that introduce interface phase and applied loads for further analyses; however, they cannot correlate with macroscopic models for trans-scale failure analysis to achieve a balance between computation costs and prediction accuracy.

Most existing studies employ stress amplification factors to establish macro–micro correlations for trans-scale failure analyses of composites, yet predominantly neglect the interfacial phase effects between the fiber and matrix. There are few studies incorporating the fiber–matrix interphase at the microscopic level to perform progressive damage analyses in the field of multi-scale modeling of composite materials.

In this study, a new RVE model considering the interface phase as a physical solid medium has been developed, and an equivalent model is adopted to characterize the mechanical properties of the interface phase. Furthermore, under the framework of macro–micro failure analyses of composite materials, the relationship between the mechanical responses of laminates at the macro scale and the fibers, matrix and interface at the micro scale has been established. Based on the micro-failure criterion, failure prediction of the fiber, matrix and interface phase can be carried out. Combined with stiffness reduction and macro–micro correlation methods, a trans-scale progressive failure analysis methodology incorporating interfacial phase effect (TFAMII) has been developed. The combined stiffness reduction and macro–micro correlation approach is used to perform the progressive failure analysis of composite materials, while the micro-failure criterion is used to predict the failure of the fibers, matrix and interface phases. An Abaqus-based tool for the rapid calculation of the strain correlation matrix at the keypoints of the micro-scale RVE is developed, and a technological method for the trans-scale failure analysis of composite materials based on a micro-scale failure criterion incorporating interface phase has been established using a subroutine technique. The proposed trans-scale failure analysis method, which introduces the interface phase for trans-scale failure analyses and can simultaneously predict the failure paths of the fibers, matrix and interface phase, can provide a new approach for the trans-scale analysis of composite materials.

## 2. Calculation of Equivalent Mechanical Properties of Interface

The transmission electron microscope analysis shows that the thickness of the interface region of carbon fiber composites is about 100–200 nm [38]. Kitchens et al. [39] proposed a comprehensive framework combining inverse analysis, numerical simulation and Bayesian inference to effectively infer the mechanical properties (thickness and modulus) of the interface region from experimental data on the macro-elastic modulus of composite materials. Jiang et al. [40] compared the interface phase thickness and properties of fast-cured and conventionally cured epoxy matrix composites in terms of quantity, and found that the interface of fast-cured epoxy composites is thinner (about 20 nm), while that of conventionally cured epoxy composites is thicker (about 40 nm). The interface thickness of different composites varies significantly, and the interface thickness of the carbon-fiber epoxy composite in this paper is taken as 100 nm. Sun et al. [41] proposed a method for computing interfacial stiffness, where the interface phase is equivalently represented as an isotropic material, and the equivalent material properties (modulus and strength) of the interphase can be calculated using the following equations:

(1) $$K_{i}=\left\{\begin{array}{c} K_{ms}+\left(K_{f}-K_{ms}\right)R\left(r\right)\;\;r_{f}<r<r_{is}\\ K_{m}+\left(K_{ms}-K_{m}\right)Q\left(r\right)\;\;r_{is}<r<r_{i}\; \end{array}\right.$$

Here, K represents the modulus E or strength σ. K_ms_ denotes the lower bound of the reasonable interfacial mechanical properties due to insufficient curing. The lower bound ratios relative to the matrix properties are $\frac{E_{ms}}{E_{m}}=0.8$, $\frac{\sigma_{ms}}{\sigma_{m}}=0.5$. It is assumed that the position of the lower bound of the interfacial mechanical properties, $r_{is}$, is located at three-quarters of the interfacial width from the fiber surface, where ${\;r}_{f}$ is the radius of the fiber, and $r_{i}$ is the outer diameter of the interface. Functions R(r) and Q(r) are constructed to match the boundary conditions:

(2) $$R\left(r\right)=\frac{1-\left(r/r_{is}\right)\exp\left(1-r/r_{is}\right)}{1-\left(r_{f}/r_{is}\right)\exp\left(1-r_{f}/r_{is}\right)}$$

(3) $$Q\left(r\right)=\frac{1-\left(r/r_{i}\right)\exp\left(1-r/r_{i}\right)}{1-\left(r_{is}/r_{i}\right)\exp\left(1-r_{is}/r_{i}\right)}$$

The average modulus or strength of the interfacial region can be expressed as follows:

(4) $$\bar{K_{i}}=\int_{r_{f}}^{r_{i}}K_{i}\left(r\right)dr/\left(r_{i}-r_{f}\right)$$

Using this method for the equivalent mechanical properties of the interface, the mechanical properties of the interface can be quickly determined once the mechanical properties of the fiber and matrix are known.

## 3. Trans-Scale Failure Analysis Method Based on New RVE Model

### 3.1. Macro–Micro Strain Transformation

**Figure 1:**
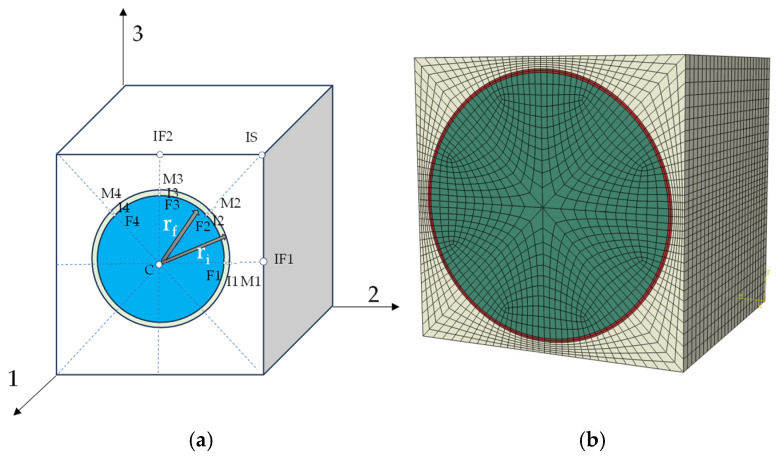
Square RVE and its key-points: (**a**) schematic of RVE and its key-points and (**b**) RVE finite element model.

The stress and strain of the fiber and matrix are the average values after they are uniformed in a finite element analysis of the composite materials with a single layer as the basic unit. However, the mechanical properties of the fiber and matrix differ significantly, resulting in the non-uniformity of the stress and strain of the fiber and matrix scale when subjected to external loads. When a failure analysis is carried out using the finite element method, it is inaccurate to judge the failure of composite materials using only the average values of uniformization. To seal the gap between the traditional RVE model and real material characteristics, a new RVE model has been developed, in which the interface phase has been introduced into the traditional RVE model to better describe the relationship between the macro scale and micro scale, under the framework of trans-scale failure analyses of composites. Based on the new RVE model, a relationship has been established between the mechanical response of the laminate at the macro scale and the fiber, matrix and interface at the micro scale, and the interface phase has been considered in the failure analysis, which is generally neglected in most studies. Strain correlation matrices were used to transform the macroscopic strain of the laminate layers into the microscopic strain of key-points in the RVE through the strain correlation matrix. Figure 1a is the square RVE considered for this study, with 1 representing the fiber direction, and 2 and 3 indicating the direction perpendicular to the fiber direction. There are 16 key-points on the RVE, which convert the macro strain to the micro strain at 16 key-points on the RVE by strain correlation. Where IF1, IF2, IS and M1-M4 denote key-points on the matrix; C and F1-F4 indicate key-points on the fiber; I1-I4 indicate key-points on the interface. The RVE model was established based on the ratio of interface thickness to fiber diameter, as shown in Figure 1b.

The transformation relationship between the ply element strain and the strain at the characteristic points of the microscopic representative volume element can be established by following formula [11]:

(5) $$\varepsilon_{i}^{k}-\alpha_{i}^{k}\Delta T=M_{ij}^{k}({\bar{\varepsilon }}_{j}-{\bar{\alpha }}_{j}\Delta T)+A_{i}^{k}\Delta T\;(i,\;j\;=\;1\sim 6)$$

where

$\varepsilon_{i}^{k}$: strain at key-point k;

${\bar{\varepsilon }}_{j}$: strain of macro model element;

$M_{ij}^{k}$: mechanical strain correlation matrices at point k in the RVE;

$A_{i}^{k}$: thermal strain correlation matrix due to deformation disharmony;

ΔT: difference between curing temperature and service temperature;

$\alpha_{i}^{k}$: coefficient of thermal expansion of matrix or fiber phase;

${\bar{\alpha }}_{j}$: equivalent thermal expansion coefficient.

Expanding on the above formula,

$${\left(\begin{array}{c} \varepsilon_{1}\\ \varepsilon_{2}\\ \varepsilon_{3}\\ \varepsilon_{4}\\ \varepsilon_{5}\\ \varepsilon_{6} \end{array}\right)}^{k}-{\left(\begin{array}{c} \alpha_{1}\\ \alpha_{2}\\ \alpha_{3}\\ \alpha_{4}\\ \alpha_{5}\\ \alpha_{6} \end{array}\right)}^{k}\Delta T={\left[\begin{array}{cccccc} M_{11} & M_{12} & M_{13} & M_{14} & M_{15} & M_{16}\\ M_{21} & M_{22} & M_{23} & M_{24} & M_{25} & M_{26}\\ M_{31} & M_{32} & M_{33} & M_{34} & M_{35} & M_{36}\\ M_{41} & M_{42} & M_{43} & M_{44} & M_{45} & M_{46}\\ M_{51} & M_{52} & M_{53} & M_{54} & M_{55} & M_{56}\\ M_{61} & M_{62} & M_{63} & M_{64} & M_{65} & M_{66} \end{array}\right]}^{k}\left(\left(\begin{array}{c} {\bar{\varepsilon }}_{1}\\ {\bar{\varepsilon }}_{2}\\ {\bar{\varepsilon }}_{3}\\ {\bar{\varepsilon }}_{4}\\ {\bar{\varepsilon }}_{5}\\ {\bar{\varepsilon }}_{6} \end{array}\right)-\left(\begin{array}{c} {\bar{\alpha }}_{1}\\ {\bar{\alpha }}_{2}\\ {\bar{\alpha }}_{3}\\ {\bar{\alpha }}_{4}\\ {\bar{\alpha }}_{5}\\ {\bar{\alpha }}_{6} \end{array}\right)\Delta T\right)+{\left(\begin{array}{c} A_{1}\\ A_{2}\\ A_{3}\\ A_{4}\\ A_{5}\\ A_{6} \end{array}\right)}^{k}\Delta T$$

This formula was originally proposed by Goose and Christensen [9] and expanded by Yudhanto [10] and Tay et al. [11]. Ha et al. [12] extended this method into a stress formula.

From the macro- and micro-strain relationships, it can be seen that to calculate the strain at the key-points of the RVE, it is necessary to first calculate the mechanical strain correlation matrix M and the thermal strain correlation matrix A. By establishing a three-dimensional finite element model of the representative elements and applying periodic boundary conditions, such as ${\bar{\varepsilon }}_{1}=1$, ${\bar{\varepsilon }}_{2}={\bar{\varepsilon }}_{3}={\bar{\varepsilon }}_{4}={\bar{\varepsilon }}_{5}={\bar{\varepsilon }}_{6}=0$, ΔT=0, one column of the mechanical strain correlation matrix can be obtained, and the corresponding periodic boundary conditions can be sequentially changed to obtain the mechanical strain correlation matrices of the remaining columns. The thermal strain correlation matrix A can also be calculated in the same way. With an external mechanical load of 0 and a given temperature change of ΔT=1, the thermal strain correlation matrix A can be calculated.

By establishing a three-dimensional finite element model of the representative elements and applying periodic boundary conditions, one column of the mechanical strain correlation matrix can be obtained, and the corresponding periodic boundary conditions can be sequentially changed to obtain the mechanical strain correlation matrices of the remaining columns. The thermal strain correlation matrix A can also be calculated in the same way. With an external mechanical load of 0 and a given temperature change, the thermal strain correlation matrix A can be calculated.

### 3.2. Trans-Scale Progressive Failure Analysis Methodology Incorporating Interfacial Phase Effect (TFAMII)

The mechanical properties of the interface between fiber and matrix are rarely considered or assumed to be an interface with zero thickness in many studies of trans-scale failure methods. In practice, the thickness of the interface is not negligible. Ignoring the interface or assuming the interface have zero thickness oversimplifies the microscopic analysis model of composite materials, which cannot properly describe its microscopic structure, and thus cannot accurately obtain results such as failure modes and ultimate loads. In this study, the interface phase was introduced into the micro-scale representative unit model, and the failures of the fibers, matrix and interface phase are discussed separately. The failures of the fibers, matrix and interface can be characterized separately. The strain correlation matrix was used to establish the trans-scale correlation relationship between the macro model and the micro model. Based on the macro-model analysis, the strain information at the key-points of the micro model was obtained, and the failure of the element was judged by the micro-scale failure criterion. The progressive failure analysis methodology for composites was developed by incorporating stiffness degradation, establishing a trans-scale failure analysis methodology that accounts for the fiber–matrix interphase effect (TFAMII).

**Figure 2:**
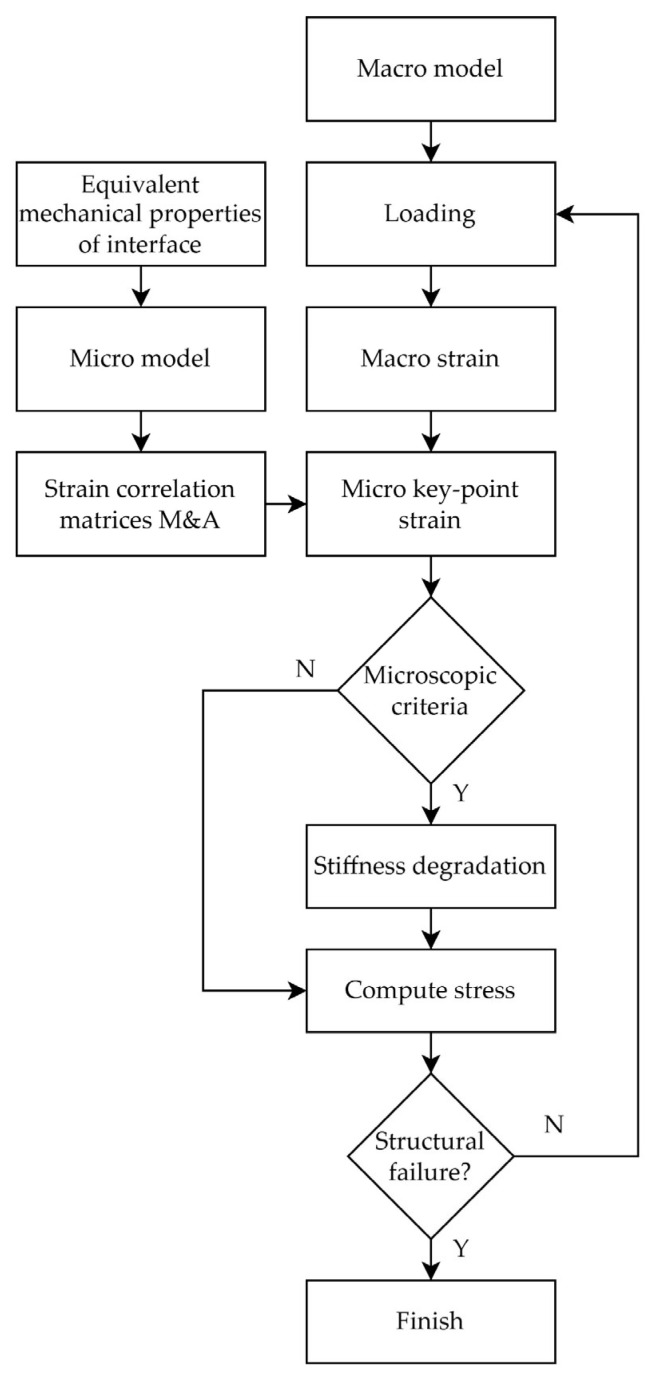
TFAMII analysis process.

The trans-scale failure analysis process is shown in Figure 2, where the equivalent mechanical properties of the interface, such as the modulus, strength, etc., were first obtained using the equivalent method for modeling the micro-scale RVE. Secondly, the refined RVE model of the composite material was established, which is accurate to the scales of the fibers, matrix and interface phase. Six different periodic boundary conditions were applied to the RVE to obtain the mechanical strain correlation matrix, and temperature loads were applied to obtain the thermal strain correlation matrix. The macro model of the composite laminate with open holes was established and the UMAT subroutine was written to verify the proposed method for the trans-scale failure analysis of composite materials incorporating interface mechanical properties. The UEXTERNALDB subroutine was employed to read the strain correlation matrix output from the microscopic RVE model calculations and transfer it to the UMAT subroutine for converting macroscopic strain into microscopic strain. The strain at the integration point of the macroscopic model was converted into the strain at the key-point on the microscopic model through the strain correlation matrix in the UMAT subroutine calculation.

The failure states of the key-points were evaluated to determine whether the corresponding macroscopic integration points should be considered failed. Upon failure detection, stiffness degradation was applied to the affected macroscopic integration points. The von Mises strain failure criterion was used to analyze the fiber failure, the first strain invariants were used to analyze the matrix failure and the von Mises strain failure criterion was used to analyze the interface failure. When any key-point satisfied the failure criterion, the corresponding fiber, matrix or interface was considered failed. The material stiffness was then degraded based on the failure states of these constituents. The analysis terminated if structural failure occurred; otherwise, the computation proceeded to the next iteration step.

### 3.3. Microscopic Failure Criteria

(1) Fiber Failure

The fiber failure criterion can be assessed using von Mises strain, which is expressed as follows:

(6) $$\varepsilon_{von}=\sqrt{\frac{1}{2}\left[{\left(\varepsilon_{1}-\varepsilon_{2}\right)}^{2}+{\left(\varepsilon_{1}-\varepsilon_{3}\right)}^{2}+{\left(\varepsilon_{2}-\varepsilon_{3}\right)}^{2}\right]}$$

where $\varepsilon_{1}$, $\varepsilon_{2}$, $\varepsilon_{3}$ are the first, second and third principal strains, respectively. The fiber failure judgment basis of the micromechanical failure criterion can be expressed as follows:

(7) $$\varepsilon_{von}^{f}\geq \varepsilon_{von,c}^{f}$$

where $\varepsilon_{von}^{f}$ indicates the von Mises strain of the fiber, and $\varepsilon_{von,c}^{f}$ represents the critical von Mises strain value of the fiber. When $\varepsilon_{von}^{f}$ exceeds $\varepsilon_{von,c}^{f}$, it indicates damage at fiber key-points in the RVE.

Defining $k_{von}=\frac{\varepsilon_{von}}{\varepsilon_{von,c}}$, for fiber failure,

(8) $$k^{f}=\max\left(k_{von}^{c},k_{von}^{F1},k_{von}^{F2},k_{von}^{F3},k_{von}^{F4}\right)$$

$k^{f}$ is the fiber failure index, and when $k^{f}$ ≥ 1, the fiber fails. When $k^{f}$ < 1, the fiber does not fail.

(2) Matrix failure

Matrix failure can be assessed using the first strain invariant. The first strain invariant can be expressed as follows:

(9) $$J_{1}=\varepsilon_{1}+\varepsilon_{2}+\varepsilon_{3}$$

The matrix failure criterion based on strain invariant failure theory can be expressed as follows:

(10) $$J_{1}^{m}\geq J_{1,c}^{m}$$

where $J_{1}^{m}$ denotes the first strain invariant of the matrix, and $J_{1,c}^{m}$ represents the critical value of the first strain invariant of the matrix. When $J_{1}^{m}$ exceeds $J_{1,c}^{m}$, it indicates damage at matrix key-points in the RVE.

Defining $k_{J}=\frac{J_{1}}{J_{1,c}}$, for matrix failure,

(11) $$k^{m}=\max\left(k_{J}^{IF1},k_{J}^{IF2},k_{J}^{IS},k_{J}^{M1},k_{J}^{M2},k_{J}^{M3},k_{J}^{M4}\right)$$

where $k^{m}$ is the matrix failure index, and when $k^{m}$ ≥ 1, the matrix fails. When $k^{m}$ < 1, the matrix does not fail.

(3) Interface failure

The interface failure can be assessed using the von Mises strain. The interface failure criterion based on the strain-invariant failure theory can be expressed as follows:

(12) $$\varepsilon_{von}^{i}\geq \varepsilon_{von,c}^{i}$$

where $\varepsilon_{von}^{i}$ indicates the von Mises strain of the interface, and $\varepsilon_{von,c}^{i}$ represents the critical von Mises strain value of the interface. When $\varepsilon_{von}^{i}$ exceeds $\varepsilon_{von,c}^{i}$, it indicates damage at interface key-points in the RVE.

Defining $k_{von}=\frac{\varepsilon_{von}}{\varepsilon_{von,c}}$, for interface failure,

(13) $$k^{i}=\max\left(k_{von}^{I1},k_{von}^{I2},k_{von}^{I3},k_{von}^{I4}\right)$$

where $k^{f}$ is the interface failure index, and when $k^{f}$ ≥ 1, the interface fails. When $k^{f}$ < 1, the interface does not fail.

### 3.4. Damage Evolution Model

When the fiber, matrix or interface meet the microscopic failure criterion, the material stiffness of the corresponding macro-damage element will gradually degrade and undergo corresponding damage evolution according to the energy-based exponential law, with the damage variables as shown in the following equation [42]:

(14) $$d_{m}=1-\frac{1}{k^{m}}e^{\left(1-k^{m}\right)\left(\frac{C_{22}\varepsilon_{22}^{t}l_{c}}{G_{m}}\right)}$$

(15) $$d_{f}=1-\frac{1}{k^{f}}e^{\left(1-k^{f}\right)\left(\frac{C_{11}\varepsilon_{11}^{t}l_{c}}{G_{f}}\right)}$$

(16) $$d_{i}=1-\frac{1}{k^{i}}e^{\left(1-k^{i}\right)\left(\frac{C_{22}\varepsilon_{22}^{t}l_{c}}{G_{m}}\right)}$$

where $d_{f}$, $d_{m}$ and $d_{i}$ are the damage variables for the fiber and matrix, respectively. The damage variables are 0 for undamaged elements and 1 for fully damaged elements. $\varepsilon_{11}^{t}$, $\varepsilon_{22}^{t}$ are the tensile failure strains of the fiber and the matrix, respectively. $G_{f}$, $G_{m}$ and $G_{i}\;$are the fracture energies of the fiber, matrix and interface, respectively. Assume $G_{m}$ = $G_{i}\;$in this paper. *l_c_* is the characteristic length used to reduce grid dependence and is related to the size of the cell. During the damage evolution stage, the stress–strain relationship of the composite is as follows:

(17) $$\sigma =C\left(d\right)\varepsilon$$

where $C\left(d\right)$ is the damage stiffness matrix, and *d* is the damage variable. $C\left(d\right)$ can be represented by the following formula:

(18) $$C(d)=\left[\begin{array}{cccccc} \left(1-d_{f}\right)C_{11} & \left(1-d_{m}\right)\left(1-d_{f}\right)C_{12} & \left(1-d_{f}\right)C_{13} & 0 & 0 & 0\\ & \left(1-d_{m}\right)C_{22} & \left(1-d_{m}\right)\left(1-d_{i}\right)C_{23} & 0 & 0 & 0\\ & & C_{33} & 0 & 0 & 0\\ & & & \left(1-d_{m}\right)\left(1-d_{i}\right)C_{44} & 0 & 0\\ & & & & C_{55} & 0\\ SYM & & & & & C_{66} \end{array}\right]$$

## 4. Example Analysis

### 4.1. Finite Element Modeling of Open-Hole Laminate

The materials selected for use in this study are T700/5428, CCF300/5428 and IM7/8552, with 63%, 63% and 60% fiber content, respectively. The thickness of a single layer is 0.125 mm, and three composite laminates selected for the study, with their dimensions and ply, are shown in Table 1. The mechanical properties of the T700/5428, CCF300/5428 and IM7/8552 composites and component materials are shown in Table 2. The mechanical properties of the fiber and matrix were used to calculate the strain correlation matrix. The mechanical properties of composite plies were used for the basic parameter inputs of the macro model.

Using the material properties of the fiber and matrix from Table 2, the equivalent mechanical properties of the interface, such as the modulus of elasticity and strength, were calculated based on the methods presented in the literature [41]. Through the representative volume element model, the strain correlation matrix can be calculated based on the mechanical properties of the fiber and matrix. The critical strain invariants for the fiber, matrix and interface phrase are shown in Table 3.

**Figure 3:**
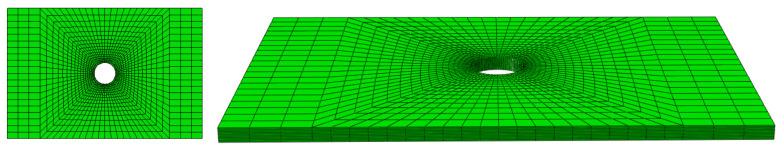
Finite element model of open-hole laminate.

A finite element model of the open-hole laminate was established, as shown in Figure 3, with a hexahedral structure grid. To improve the accuracy of the calculation results, the mesh of the perforated section in the center of the sample was encrypted, with one element for each layer in the thickness direction. Considering the symmetry relationship, only half of the ply needs to be established in the finite element model. For the tensile failure analysis of an open-hole laminate, one end of the laminate was fixed and a displacement load was applied at the other end.

### 4.2. Results and Discussion

**Figure 4:**
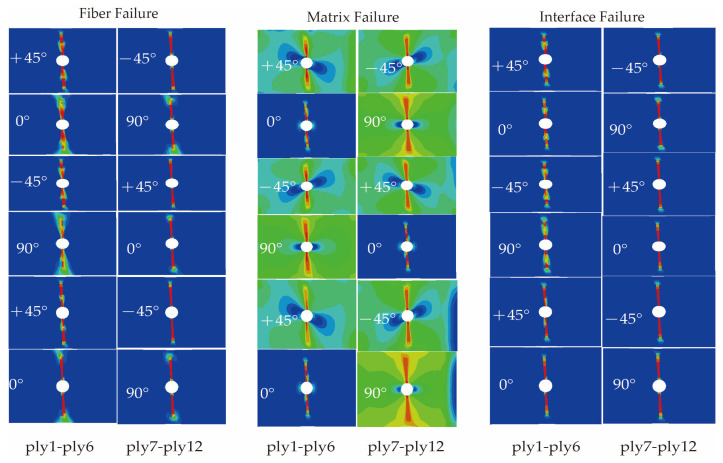
Predicted damage patterns of T700/5428 ([45/0/−45/90]_3s_) open-hole laminate.

**Figure 5:**
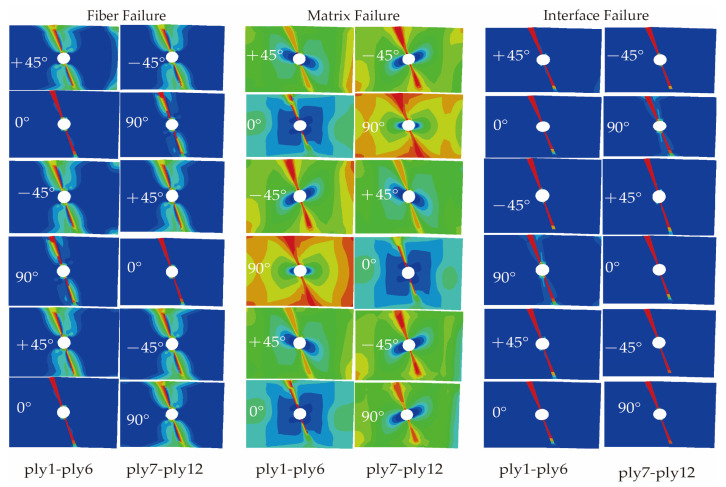
Predicted damage patterns of CCF300/5428 ([45/0/−45/90]_3s_) open-hole laminate.

**Figure 6:**
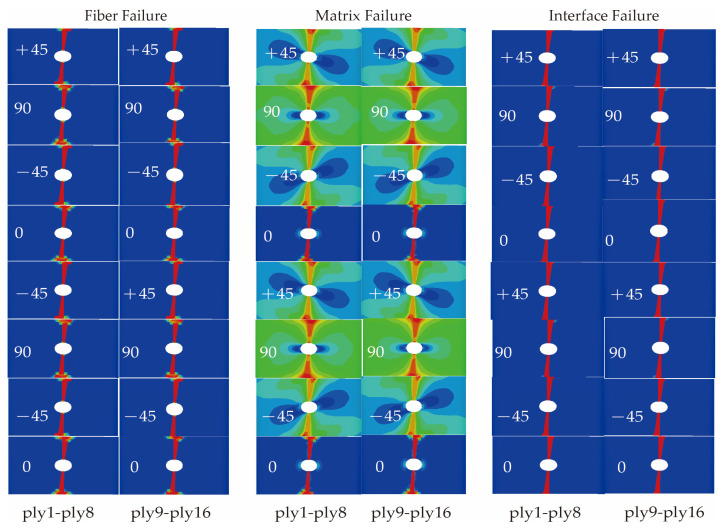
Predicted damage patterns of IM7/8552 ([45/90/−45/0]_4s_) open-hole laminate.

Based on the proposed trans-scale progressive failure analysis methodology incorporating the interfacial phase effect (TFAMII), the strain correlation matrix was used to convert the macro strain at a macro-scale to the micro strain of the key-points at a micro-scale to assess the onset of damage. Combined with the stiffness degradation method, the ultimate strength of the composite laminate under tensile loading was calculated. During the loading process, the stress concentration around the open hole leads to damage initiation, followed by damage evolution, and the damage variable increases gradually. The predicted damage modes for the three types of material simulations are shown in Figure 4, Figure 5 and Figure 6, respectively. Compared with previous studies [23,29,47], the method proposed in this study can not only observe the failure path of the fiber and matrix on the macro-failure contours, but also additionally observe the failure path of the interface phase. There are significant differences in failure behavior among the three material systems, consistent with the experimental results reported in the literature [21,43]. The T700/5428 matrix type is identical to that of CCF300/5428, and it exhibits a higher fiber tensile strength. The high strength of the open-hole laminate is primarily attributed to the fiber. Widespread matrix failure was observed in the ±45° and 90° layups, while fiber failure primarily occurred in the 0°, ±45° and inner 90° layups. Falzon and Chamis et al. [48,49] mentioned that the Poisson’s ratio has a certain effect on the stress in the 90° layer. In the 90° layer, there is some fiber compression failure near the hole due to the Poisson’s ratio effect. The failure path at the interface is the same as that of the fiber, with the inner layer suffering more severe damage than the outer layer. Fiber failure in the CCF300/5428 composite primarily occurs in the 0° layup, and the failure path is not perpendicular to the long axis of the composite, which is consistent with the experimental observations in the literature. Compared to T700/5428, in all layups, more severe matrix failure was observed before the final failure. In the ±45° layers, the contour of the matrix failure was parallel to the fiber direction, and the failure path at the interface was the same as that of the fiber. In the ±45° and 90° layers, there was minor fiber damage due to the Poisson’s ratio effect. Fiber damage was observed in all layups of the IM7/8552 composite material, primarily due to the higher Poisson’s ratio effect of CCF300/5428 compared to T700/5428. CCF300/5428 has the smallest Poisson’s ratio coefficient, resulting in fewer fiber failures in the ±45° and 90° layers due to the Poisson’s ratio effect. The failure paths of the matrix, interface and fiber for all three materials are consistent with the fiber failure paths.

**Figure 7:**
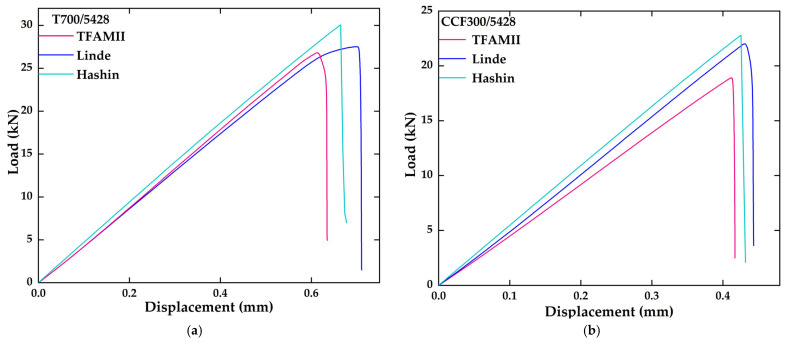

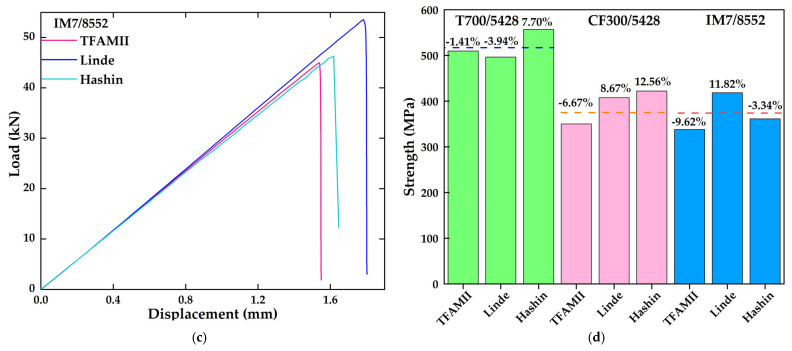
Comparison of tensile load–displacement curves and ultimate strength: (**a**) T700/5428; (**b**) CCF300/5428; and (**c**) IM7/8552. (**d**) Ultimate tensile strength from TFAMII, Linde and experimental methods (the dashed lines represent the corresponding experimental results).

The Hashin failure criterion and Linde failure criterion [42] are macroscopic failure criteria that distinguish fiber failure and matrix failure, and are used as a comparison object for the TFAMII. A comparison of the load–displacement curves obtained by the TFAMII method with those calculated by the Linde method and Hashin method is shown in Figure 7a–c and the ultimate tensile strength from the TFAMII, Linde, Hashin and experimental methods is shown in the Figure 7d. They all agree well with the experimental results. It can be observed that the load–displacement curve remains relatively flat prior to failure caused by fiber breakage, indicating that matrix damage does not significantly reduce the stiffness of the laminate. The slope is highest for IM7/8552 and lowest for T700/5428, due to the differing tensile moduli of CCF300, T700 and IM7 fiber. When the final failure occurs, all three curves drop sharply. For the T700/5428 open-hole laminate, the ultimate strength obtained by the trans-scale method incorporating interface phase effects is 509.7 MPa, with a relative error of −1.41% compared to the experimentally obtained ultimate strength of 517 MPa [21]; The Linde and Hashin failure method yielded an ultimate strength of 496.5 MPa and 556.8, respectively, with a relative error of −3.97% and 7.70%. For the CCF300/5428 open-hole laminate, the ultimate strength obtained by the trans-scale method incorporating the interface phase effect is 350.0 MPa [21], with a relative error of −6.67% compared to the experimentally obtained ultimate strength of 375 MPa. Ultimate strengths of 407.5 MPa and 442.6 MPa with a relative error of 8.67% and 12.56%, respectively, were obtained by the Linde and Hashin failure methods. The ultimate strength obtained for IM7/8552 by the trans-scale method incorporating interface phase effects is 338.0 MPa [43], with a relative error of −9.62% from the experimentally obtained ultimate strength of 374 MPa. Ultimate strengths of 418.2 MPa and 361.5 MPa, with a relative error of 11.82% and −3.34%, respectively, were obtained from the Linde and Hashin failure methods. The error between the TFAMII results and experimental values for all three different composites is less than 10%. A comparison of the ultimate tensile strengths obtained from the proposed TFAMII method, Linde method, Hashin method and experimental results are shown in Table 4.

The numerical prediction results indicate that the proposed trans-scale failure analysis methodology can effectively analyze the failure behavior of composite laminates.

## 5. Conclusions

In this study, a trans-scale failure analysis method incorporating interface phase effects (TFAMII) for fiber-reinforced polymer matrix composites has been developed, which can be used to assess failure based on the micro-scale mechanical response information of fibers, matrix and interfaces. By establishing an equivalent mechanical property characterization model that considers the interfacial phase characteristics between fiber and matrix, the interfacial phase effect is introduced into the trans-scale failure analysis framework of composite materials successfully. Based on the micro-failure criterion, failure predictions of fibers, matrix and interface phases can be performed. Moreover, combined with stiffness reduction and macro–micro correlation methods, trans-scale progressive failure analysis of composite materials can be carried out. The current proposed trans-scale progressive failure analysis methodology incorporating interfacial phase effect trans-scale failure method is only suitable for analysis of unidirectional tape composite laminates, future efforts should be taken to expand the scope of application and enhancing universality are the future development directions for this trans-scale failure analysis method. Exploring failure prediction for woven composites, and for more complex structures, such as stiffened panels, will broaden the applicability of this method beyond failure predictions for composite multi-directional laminates.

Based on the proposed TFAMII method, numerical simulations of the tensile properties of typical open-hole tension laminates were performed, and compared with experimental and other methods. The effectiveness of the proposed method has been verified by comparing the predicted data, Linde failure criterion prediction data and experimental data. The method developed in this study can provide new ideas for the progressive failure analysis of composite materials.

## Figures and Tables

**Table 1:** Dimensions of composite laminate [21,43].

Materials Name	Layers	Length/mm	Width/mm	Hole Diameter/mm
T700/5428	[45/0/−5/90]_3s_	54	36	6
CCF300/5428	[45/0/−45/90]_3s_	54	36	6
IM7/8552	[45/90/−45/0]_4s_	254	64	12.7

**Table 2:** Mechanical properties of materials [21,30,35,44,45].

Materials Name	E_11_ (GPa)	E_22_ = E_33_ (GPa)	G_12_ = G_13_ (GPa)	G_23_ (GPa)	*v*_12_ = *v*_13_	*v*_23_	$\alpha_{11}({℃}^{-1})$	$\alpha_{22}({℃}^{-1})$
T700	230	18.2	36.2	7	0.27	0.3	−5.4 × 10^−7^	1.01 × 10^−5^
CCF300	252	25.2	7	6.9	0.279	0.25	1.3 × 10^−7^	1 × 10^−5^
IM7	276	19	27	7	0.2	0.247	−4 × 10^−7^	5.6 × 10^−6^
5428	3.5		/		0.35		4.41 × 10^−5^	
8552	4.08		/		0.38		4.67 × 10^−5^	
T700/5428	125	7.8	5.6	5.7	0.32	0.46	9.7 × 10^−7^	2.09 × 10^−5^
CCF300/5428	145	9.75	5.69	5.69	0.312	0.44	4 × 10^−7^	2.5 × 10^−5^
IM7/8552	165	9	5.6	2.8	0.34	0.5	−1 × 10^−6^	1.8 × 10^−5^

**Table 3:** Critical strain invariants.

Material Names	Critical Strain Invariants	Value
T700/5428 [46]	$\varepsilon_{von,c}^{f}$	0.023
	$J_{1,c}^{m}$	0.0313
	$\varepsilon_{von,c}^{i}$	0.036
CCF300/5428 [46]	$\varepsilon_{von,c}^{f}$	0.018
	$J_{1,c}^{m}$	0.0319
	$\varepsilon_{von,c}^{i}$	0.0359
IM7/8552	$\varepsilon_{von,c}^{f}$	0.0177
	$J_{1,c}^{m}$	0.0225
	$\varepsilon_{von,c}^{i}$	0.0345

**Table 4:** Comparison of ultimate tensile strength from TFAMII, Linde and experimental methods.

Materials Name	Method	Ultimate Strength/MPa	Error/%
T700/5428	Experiment [21]	517	/
	TFAMII	509.7	−1.41
	Linde	496.5	−3.97
	Hashin	556.8	7.70
CCF300/5428	Experiment [21]	375	/
	TFAMII	350.0	−6.67
	Linde	407.5	8.67
	Hashin	422.6	12.56
IM/78552	Experiment [43]	374	/
	TFAMII	338.0	−9.62
	Linde	418.2	11.82
	Hashin	361.5	−3.34

## Data Availability

The original contributions presented in the study are included in the article. Further inquiries can be directed to the corresponding author.
